# Birth weight and cardiac function assessed by echocardiography in adolescence: Avon Longitudinal Study of Parents and Children

**DOI:** 10.1002/uog.20128

**Published:** 2019-08-05

**Authors:** S. Timpka, A. D. Hughes, N. Chaturvedi, P. W. Franks, D. A. Lawlor, J. W. Rich‐Edwards, A. Fraser

**Affiliations:** ^1^ Genetic and Molecular Epidemiology Unit Lund University Diabetes Centre, Lund University Malmö Sweden; ^2^ Connors Center for Women's Health and Gender Biology Brigham and Women's Hospital and Harvard Medical School Boston MA USA; ^3^ Institute of Cardiovascular Science University College London London UK; ^4^ Harvard T. H. Chan School of Public Health Harvard University Boston MA USA; ^5^ Department of Population Health Sciences, Medical School University of Bristol Bristol UK; ^6^ MRC Integrative Epidemiology Unit at the University of Bristol University of Bristol Bristol UK; ^7^ NIHR Biomedical Research Centre at the University Hospitals Bristol NHS Foundation Trust and the University of Bristol Bristol UK

**Keywords:** ALSPAC, cardiac development, echocardiography, epidemiology, fetal growth restriction

## Abstract

**Objective:**

Maternal hemodynamics in pregnancy is associated with fetal growth and birth weight, which in turn are associated with offspring cardiovascular disease later in life. The aim of this study was to quantify the extent to which birth weight is associated with cardiac structure and function in adolescence.

**Methods:**

A subset of offspring (*n* = 1964; 55% female) of the Avon Longitudinal Study of Parents and Children were examined with echocardiography at a mean age of 17.7 (SD, 0.3) years. The associations of birth‐weight *Z*‐score for sex and gestational age with cardiac structure (assessed by relative wall thickness, left ventricular mass index (LVMI) and left atrial diameter index), systolic function (assessed by ejection fraction and left ventricular wall velocity) and diastolic function (assessed by early/late mitral inflow velocity (E/A) and early mitral inflow velocity/mitral annular early diastolic velocity (E/e′)) were evaluated. Linear regression models were adjusted for several potential confounders, including maternal prepregnancy body mass index, age, level of education and smoking during pregnancy.

**Results:**

Higher birth‐weight *Z*‐score was associated with lower E/A (mean difference, −0.024; 95% CI, −0.043 to −0.005) and E/e′ (mean difference, −0.05; 95% CI, −0.10 to −0.001) and higher LVMI (mean difference, 0.38 g/m^2.7^; 95% CI, 0.09 to 0.67). There was no or inconsistent evidence of associations of birth‐weight *Z*‐score with relative wall thickness, left atrial diameter and measurements of systolic function. Further analyses suggested that the association between birth‐weight *Z*‐score and LVMI was driven mainly by an association observed in participants born small‐for‐gestational age and it did not persist when risk factors in adolescence were accounted for.

**Conclusions:**

Higher birth weight adjusted for sex and gestational age was associated with differences in measures of diastolic function in adolescence, but the observed associations were small. It remains to be determined the extent to which these associations translate into increased susceptibility to cardiovascular disease later in life. © 2018 The Authors. *Ultrasound in Obstetrics & Gynecology* published by John Wiley & Sons Ltd on behalf of the International Society of Ultrasound in Obstetrics and Gynecology.

## INTRODUCTION

Building on the initial work by Forsdahl^1^ and Barker *et al*.^2^, epidemiological and animal studies suggest that the intrauterine environment and early‐life development are potentially important determinants of cardiovascular disease (CVD) later in life^3^. For example, a hypoxic intrauterine environment results in fetal growth restriction (FGR)^4^ and lower birth weight, which might affect fetal cardiovascular development and increase the risk of CVD in adulthood^5^, ^6^, ^7^, ^8^, ^9^.

Studies in young children suggest that FGR, defined as being born small‐for‐gestational age (SGA) with additional evidence of placental dysfunction, is associated with altered cardiac development^10^, ^11^. Similar, but more modest, associations have been observed in those born SGA without evidence of placental dysfunction^12^, ^13^, ^14^, ^15^. In adults, evidence of an association between birth weight and cardiac structure is contradictory or absent^16^, ^17^, ^18^, but most studies did not account for gestational age at birth^18^ or were restricted to those born at term^17^. Furthermore, there is an intricate relationship between fetal growth and cardiovascular hemodynamics^19^ that might result in increased susceptibility to age‐related risk factors, such as blood pressure, later in life. Studies on the association between fetal growth and cardiac development from early childhood to middle age are thus needed. Myocardial differences observed in childhood could be abrogated in adulthood through compensatory mechanisms but still translate into worse cardiovascular outcome if the compensation is through tissue growth^20^ at the expense of cardiac function.

In this study, we aimed to investigate the association between birth weight (adjusted for sex and gestational age) and cardiac structure and function in adolescence, focusing on a set of complementary echocardiographic outcomes, each denoting different aspects of left atrial and ventricular structure and function, in a birth cohort based in the UK.

## METHODS

**Figure 1:**
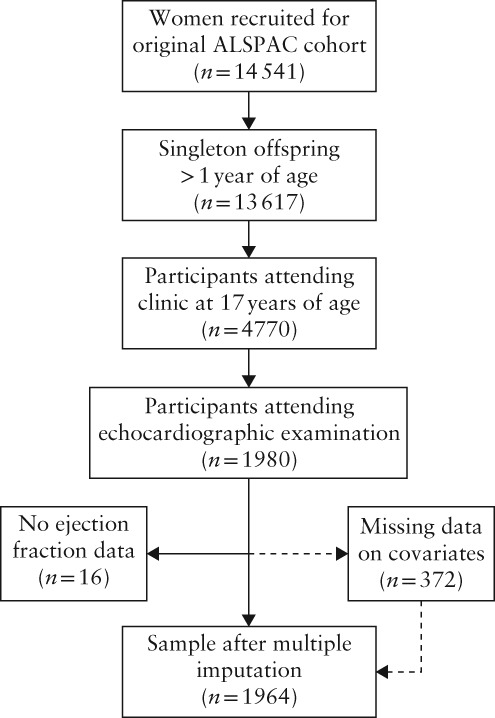
Flowchart summarizing inclusion in study of subset of offspring in Avon Longitudinal Study of Parents and Children (ALSPAC)

This study includes a subset of offspring of the Avon Longitudinal Study of Parents and Children (ALSPAC), a prospective population‐based birth cohort. Pregnant women resident in the Bristol area of South West England, UK, with expected dates of delivery between 1 April 1991 and 31 December 1992, were eligible for recruitment. Of 14 541 women originally recruited, there were 13 617 singleton offspring alive at > 1 year of age. In total, 4770 of these participated in a clinic assessment at 17 years of age and a random subsample was examined with echocardiography (*n* = 1980). Those with a successful examination, defined in this study as having an estimable ejection fraction, were included in the study sample (*n* = 1964; Figure 1). The study website contains details of all the data that are available through a fully searchable data dictionary (http://www.bristol.ac.uk/alspac/researchers/our‐data/). Ethical approval for the study was obtained from the ALSPAC Ethics and Law Committee and the Local Research Ethics Committees. Informed consent for the use of data collected via questionnaires and clinics was obtained from participants following the recommendations of the ALSPAC Ethics and Law Committee at the time.

### Ascertainment of birth weight and covariables

Data collection in ALSPAC has been described in detail for mothers^21^ and offspring^22^ elsewhere. In summary, data on birth weight, gestational age at birth and maternal blood pressure, as well as urine dipstick measurements during pregnancy, were extracted from medical records by six trained midwives. Birth weight was standardized as a birth‐weight *Z*‐score for gestational age by sex, using an external and recently updated birth‐weight reference based on UK data from the same time period as that in which the participants were born (early 1990s)^23^. Additional information on data collection methods and definitions can be found in Appendix S1.

### Echocardiographic examination

The echocardiography examination protocol has been described previously^24^. One of two experienced echocardiographers performed the examinations following a standard protocol using an HDI 5000 ultrasound machine (Philips Medical Systems, North Andover, MA, USA) equipped with a P4‐2 phased‐array ultrasound transducer. All measurements were performed according to the American Society of Echocardiography guidelines. Based on previous literature and to limit multiple comparisons, seven echocardiographic measurements were focused on as outcomes: three were principally indicators of cardiac structure (relative wall thickness (RWT), left ventricular mass indexed to height in m^2.7^ (LVMI)^25^ and left atrial diameter indexed to height in m (LADI)); two for systolic function (ejection fraction and left ventricular wall velocity as measured with tissue Doppler (s′)); and two for diastolic function (early/late mitral inflow velocity (E/A) and early mitral inflow velocity/mitral annular early diastolic velocity (E/e′)). The echocardiographic examinations are described further in Appendix S1.

### Statistical analysis

We first tested the extent to which modeling birth‐weight *Z*‐score as a continuous variable was appropriate compared with different categorizations of birth‐weight *Z*‐score that would allow for a non‐linear association across birth‐weight distribution. To do so, model‐fit statistics of a model with continuous birth‐weight *Z*‐score were compared with models including birth‐weight *Z*‐score categorized in various ways for each echocardiography outcome (Appendix S1).

In Model I, we adjusted for sex and offspring age at the clinical assessment. In the main confounder‐adjusted model (Model II), we adjusted further for maternal height, maternal education and pregnancy‐related factors including maternal age, parity, maternal prepregnancy body mass index (BMI), maternal diabetes mellitus/glycosuria during pregnancy, preterm birth, hypertensive disorders of pregnancy and maternal smoking during pregnancy. These confounders were chosen *a priori* as variables that are plausible shared antecedent causes of the exposure (birth‐weight *Z*‐score) and the cardiac outcomes. In Model III, we adjusted further for participant factors in adolescence, including BMI, systolic blood pressure (SBP) and heart rate, all of which are potential mediators of the association between birth weight and cardiac outcome. Consequently, the results of this model might be interpreted as indicating the effect of birth weight once the effects of birth weight acting through these mediators is accounted for; however, mediation models have considerable potential to introduce other biases and the resulting model should be interpreted with caution and in the context of the assumptions made^26^.

All analyses were repeated in participants with complete data on birth weight and covariables (*n* = 1592). To test the extent to which any observed association was also present in those with normal birth‐weight *Z*‐score, the analyses were repeated but restricting the sample to those with birth‐weight *Z*‐score between the 10^th^ and 90^th^ percentiles (thus excluding those with either small or large birth weight for gestational age and sex). To investigate the extent to which the indexing strategy influenced our results for LVMI and LADI, we performed complementary analyses in which we used different strategies to account for participant height (Appendix S2). Additionally, interaction analyses were performed according to sex, as there have been reports of different responses to adverse fetal environment between the sexes^27^.

### Multiple imputation

In total, 372 (18.9%) included participants had missing data for birth weight or other covariables. To increase power and minimize bias, we used multiple imputation to impute missing values of birth weight and covariables for all participants. All imputed variables had < 10% missing data. Twenty imputed datasets were generated and analyzed by implementing the ‘mi impute’ and ‘mi estimate’ commands in Stata 13.1 (StataCorp LLC, College Station, TX, USA; Appendix S1). Data for males and females were imputed separately to allow for testing of sex interactions in the imputed datasets.

## RESULTS

Mean birth weight in our sample was 3.42 (SD, 0.52) kg. Characteristics of the study sample and of other singleton participants in ALSPAC attending the visit at 17 years of age are shown in Table 1. Mean birth‐weight *Z*‐score for gestational age and sex was similar between the two groups. Using model‐fit statistics, no strong evidence to suggest non‐linearity in the associations between birth‐weight *Z*‐score and outcome was observed (Appendix S2).

**Table 1:** Characteristics of study sample and other singleton participants of Avon Longitudinal Study of Parents and Children (ALSPAC) attending clinical assessment at 17 years of age

Characteristic	Study sample (*n* = 1964)		Other ALSPAC participants (*n* = 2806)	
	Value	*n*	Value	*n*
*Maternal/pregnancy*				
Female offspring	1080 (55.0)	1964	1596 (56.9)	2806
Birth weight (kg)	3.42 ± 0.52	1940	3.44 ± 0.53	2777
Birth‐weight *Z*‐score	0.12 ± 1.00	1940	0.15 ± 1.09	2776
Maternal age at delivery (years)	29.4 ± 4.6	1964	29.0 ± 4.7	2806
Maternal height (m)	1.64 ± 0.07	1868	1.64 ± 0.07	2661
Maternal prepregnancy BMI (kg/m^2^)	22.2 (20.5–24.4)	1776	22.0 (20.5–24.2)	2554
First pregnancy	955 (50.0)	1911	1268 (46.9)	2701
Preterm birth (< 37 weeks)	85 (4.3)	1964	126 (4.5)	2806
Diabetes/glycosuria during pregnancy	68 (3.6)	1890	121 (4.5)	2693
Maternal HDP or hypertension		1870		2676
No HDP or hypertension	1474 (78.8)		2139 (79.9)	
Gestational hypertension	294 (15.7)		379 (14.2)	
Pre‐eclampsia	49 (2.6)		58 (2.2)	
Essential hypertension	53 (2.8)		100 (3.7)	
Maternal smoking status during pregnancy		1928		2746
Never smoked	1527 (79.2)		2068 (75.3)	
Stopped prior to second trimester	173 (9.0)		313 (11.4)	
Smoked during second trimester	228 (11.8)		365 (13.3)	
Maternal educational level		1906		2698
Compulsory/vocational	375 (19.7)		521 (19.3)	
Compulsory/higher achievement	637 (33.4)		934 (34.6)	
Secondary/academic preparation	508 (26.7)		769 (28.5)	
Tertiary/degree	386 (20.3)		474 (17.6)	
*17‐year follow‐up*				
Offspring age (years)	17.7 ± 0.3	1964	17.9 ± 0.5	2806
BMI (kg/m^2^)	21.9 (20.1–24.7)	1929	21.9 (20.1–24.5)	2703
Heart rate (bpm)	64 ± 10	1905	65 ± 10	2392
Systolic blood pressure (mmHg)	119 ± 11	1905	118 ± 11	2392
Diastolic blood pressure (mmHg)	64 ± 6	1905	64 ± 7	2392
Cardiac structure				
LVMI (g/m^2.7^)	28.9 ± 6.2	1929	—	—
LADI (cm/m)	1.86 ± 0.23	1730	—	—
Relative wall thickness	0.38 ± 0.06	1963	—	—
Systolic function				
Ejection fraction (%)	66.8 ± 6.4	1964	—	—
Average s′ (cm/s)	7.8 ± 1.4	1881	—	—
Diastolic function				
E/A ratio	1.93 ± 0.40	1895	—	—
E/e′ ratio	4.9 ± 1.0	1878	—	—

Data are given as *n* (%), mean ± SD or median (interquartile range).

BMI, body mass index; E/A, early/late mitral inflow velocity; E/e′, early mitral inflow velocity/mitral annular early diastolic velocity; HDP, hypertensive disorder of pregnancy; LADI, left atrial diameter indexed to height in m; LVMI, left ventricular mass indexed to height in m^2.7^; s′, left ventricular wall velocity measured with tissue Doppler.

Table 2 shows the linear association between birth‐weight *Z*‐score and measures of cardiac structure and function in adolescence. In the main model adjusted for maternal and pregnancy factors as well as offspring sex and age (Model II), higher birth‐weight *Z*‐score was associated with larger LVMI and differences in diastolic function as assessed by E/A and E/e′. Additional adjustment for factors in adolescence (BMI, SBP and heart rate; Model III) substantially attenuated the association between birth‐weight *Z*‐score and LVMI, and evidence of an inverse association with LADI emerged. There was no evidence of associations with RWT or ejection fraction.

**Table 2:** Association between birth‐weight *Z*‐score and cardiac structure and function at 17 years of age

Model*	Cardiac structure			Systolic function		Diastolic function	
	LVMI (g/m^2.7^) (*n* = 1929)	LADI (cm/m) (*n* = 1730)	RWT (*n* = 1963)	EF (%) (*n* = 1964)	s′ (cm/s) (*n* = 1881)	E/A ratio (*n* = 1895)	E/e′ ratio (*n* = 1878)
I	0.38 (0.11 to 0.66)	−0.002 (−0.013 to 0.009)	0.001 (−0.002 to 0.003)	−0.22 (−0.50 to 0.06)	0.06 (−0.0003 to 0.13)	−0.028 (−0.046 to −0.010)	−0.06 (−0.10 to −0.01)
II	0.38 (0.09 to 0.67)	−0.007 (−0.018 to 0.005)	0.001 (−0.001 to 0.004)	−0.23 (−0.53 to 0.07)	0.05 (−0.02 to 0.12)	−0.024 (−0.043 to −0.005)	−0.05 (−0.10 to −0.001)
III	0.08 (−0.17 to 0.32)	−0.017 (−0.028 to −0.007)	0.001 (−0.002 to 0.004)	−0.20 (−0.50 to 0.10)	0.05 (−0.02 to 0.12)	−0.026 (−0.045 to −0.007)	−0.04 (−0.09 to 0.01)

Data are mean difference per 1 SD (corresponds roughly to 0.45 kg at 40 weeks' gestation) in birth weight (95% CI).

* Birth weight modeled as standardized by gestational week and sex.

Model I, adjusted for age at examination and sex (male or female); Model II, adjusted additionally for maternal height, maternal hypertensive disorders of pregnancy (pre‐eclampsia, gestational hypertension, essential hypertension or none), maternal age at pregnancy, maternal body mass index (BMI), maternal parity (parous or nulliparous), maternal smoking status during pregnancy (never, stopped prior to second trimester or smoked during second trimester), maternal education (compulsory/vocational, compulsory/higher achievement, secondary/academic preparation or tertiary/degree), maternal diabetes mellitus/glycosuria during pregnancy (yes or no) and preterm birth (< 37 weeks; yes or no); Model III, adjusted additionally for factors in adolescence: BMI, systolic blood pressure and heart rate.

E/A, early/late mitral inflow velocity; E/e′, early mitral inflow velocity/mitral annular early diastolic velocity; EF, ejection fraction; LADI, left atrial diameter indexed to height in m; LVMI, left ventricular mass indexed to height in m^2.7^; RWT, relative wall thickness; s′, left ventricular wall velocity measured with tissue Doppler.

Table 3 shows the associations between birth‐weight *Z*‐score and echocardiography outcome in participants with normal birth‐weight *Z*‐score (between the 10^th^ and 90^th^ percentiles). Among these participants, there was no evidence of an association between birth‐weight *Z*‐score and LVMI. However, the estimates of inverse associations with diastolic function (E/A and E/e′) were similar to those observed in the main analysis. To explore these findings further, we generated linear splines with knots at the 10^th^ and 90^th^ percentiles of birth‐weight *Z*‐score (Appendix S2). This analysis revealed a positive association between birth‐weight *Z*‐score and LVMI (mean difference, 2.44; 95% CI, 0.89–4.00) that was restricted to participants with low birth weight (*Z*‐score < 10^th^ percentile). Restricting analyses to participants with no missing data provided similar results compared to the main analyses (Appendix S2, Tables S1 and S2). In the complementary analyses accounting for height, the results for LVMI remained similar, while the associations for left atrial size appeared somewhat strengthened in Model II (Appendix S2 and Table S3). We did not observe any strong evidence of an interaction of sex for any outcome (data not shown). The most discordant analysis between female and male participants was the association between birth weight and s′, which was observed in females (mean difference, 0.10 cm/s; 95% CI, 0.01–0.20) but not in males (mean difference, −0.01 cm/s; 95% CI, −0.11 to 0.10); *P* = 0.06 for interaction.

**Table 3:** Association between birth‐weight *Z*‐score and cardiac structure and function at 17 years of age in participants with normal birth weight (*Z*‐score between 10^th^ and 90^th^ percentiles)

Parameter	Difference per 1 SD (95% CI)*
Cardiac structure	
LVMI (g/m^2.7^)	0.01 (−0.45 to 0.48)
LADI (cm/m)	−0.007 (−0.026 to 0.012)
Relative wall thickness	−0.0002 (−0.005 to 0.004)
Systolic function	
Ejection fraction (%)	−0.45 (−0.93 to 0.03)
s′ (cm/s)	0.11 (−0.004 to 0.22)
Diastolic function	
E/A ratio	−0.03 (−0.07 to −0.003)
E/e′ ratio	−0.08 (−0.16 to −0.001)

Number of participants with available outcome data varies by echocardiographic measurement; *n* is allowed to vary in each imputed dataset as only participants with normal birth weight are included in analyses.

Birth weight modeled as standardized by gestational age and sex.

Model II, adjusted for age at examination, sex (male or female), maternal height, maternal hypertensive disorders of pregnancy (pre‐eclampsia, gestational hypertension, essential hypertension or none), maternal age at pregnancy, maternal body mass index, maternal parity (parous or nulliparous), maternal smoking status during pregnancy (never, stopped prior to second trimester, smoked during second trimester), maternal education (compulsory/vocational, compulsory/higher achievement, secondary/academic preparation or tertiary/degree), maternal diabetes mellitus/glycosuria during pregnancy (yes or no) and preterm birth (< 37 weeks; yes or no).

* 1 SD (in birth weight) corresponds roughly to 0.45 kg at 40 weeks' gestation.

E/A, early/late mitral inflow velocity; E/e′, early mitral inflow velocity/mitral annular early diastolic velocity; LADI, left atrial diameter indexed to height in m; LVMI, left ventricular mass indexed to height in m^2.7^; s′, left ventricular wall velocity measured with tissue Doppler.

## DISCUSSION

In this study, we found higher birth weight adjusted for sex and gestational age to be associated with measures of cardiac diastolic function in late adolescence. We also observed a positive linear association between birth‐weight *Z*‐score and LVMI, but this appeared to be driven by participants born SGA. The observed effect sizes are small, corresponding to roughly 1–2% of the mean echocardiography measurement in the sample per 1 SD of birth weight, and should not have clinical relevance in young adulthood. The novelty and potential clinical implications of the results might be best understood if viewed from a life‐course perspective. During gestation and throughout life, cardiac and vascular structures develop and function interdependently. Verburg *et al*.^19^ reported that FGR, defined by ultrasound and placental function, is associated with adaptive cardiovascular changes in the fetus. Furthermore, the number of cardiomyocytes at birth appears to determine the number of cells later in life^20^. Subsequent growth of the tissue is mediated mostly through increased cellular size and not numbers. Thus, the trajectories of cardiac growth and development over the life course might be dependent on prenatal factors that are reflected in birth weight. In middle age, greater left ventricular mass is a risk factor for coronary heart disease^28^ and larger left ventricular mass in older adults is associated with developing reduced ventricular function^29^. Consequently, a model of increased susceptibility with age in those with low birth weight might be accurate; the first insult of reduced fetal growth results in suboptimal myocardial development, which translates into increased susceptibility to age‐related increase in afterload^30^ and CVD risk‐factor burden. Another potential explanation for our results is that there are pleiotropic genetic effects that influence both fetal growth and cardiac structure and function. Investigations on the extent to which trajectories of cardiac remodeling across the life course differ according to birth‐weight status are needed and would add substantially to our understanding of cardiac development and adaptation.

Crispi *et al*.^10^ and Sarvari *et al*.^11^ reported FGR (defined as low birth weight and evidence of placental dysfunction) to be associated with a more globular‐shaped heart in preschool children and preadolescents. Geelhoed *et al*.^15^ reported that higher birth weight was associated with higher left ventricular mass at 2 years of age, while Hietalampi *et al*.^14^ observed an association between birth weight and left ventricular mass at the age of 15 years. Although we initially found support for a similar association in this study, further analyses suggested that the association between birth weight and LVMI was restricted to participants born SGA. Birth weight is associated with blood pressure^31^, BMI^32^ and heart rate^33^ later in life and we considered these variables as potential mediators in the analyses. Still, only the effect estimates for LVMI and LADI appeared quantitatively different in the model that included these mediators in adolescence (Model III) compared to our main confounder‐adjusted model (Model II). These differences could potentially be explained by a large proportion of the association being mediated by the variables included additionally or the presence of collider‐stratification bias^26^.

This study also suggests that greater birth weight for gestational age might be associated with relatively smaller left atrial size. Though this observation was dependent on the handling of height in the models, the most plausible models all supported an association. A larger left atrial size in general is associated with increased risk of atrial fibrillation^34^ and CVD events^35^ in adults. However, there is conflicting evidence of the association between birth weight and atrial fibrillation^36^, and the use of crude birth weight without adjusting for gestational age and the potential for adult height to mediate some of the effect contribute further to the difficult interpretation of these studies.

E/e′ correlates with the left ventricular diastolic filling pressure (in general, lower is better), predicts cardiac events among patients with hypertension^37^ and is one of several measurements recommended to evaluate diastolic dysfunction in heart failure diagnosis^38^, although its validity as a measure of left atrial filling pressure in healthy individuals is controversial^39^. Crispi *et al*.^10^, ^12^ reported that FGR was associated with smaller s′ and larger E/e′ in 5‐year‐old children, with a similar but attenuated association observed in offspring born SGA. Our results suggest that, in ALSPAC, altered diastolic function is not confined to those born with lower birth weight, but there is a negative association between birth weight and diastolic function across the birth‐weight distribution. s′, a sensitive marker of systolic left ventricular function, is associated negatively with risk of heart failure and cardiovascular death, independent of classical cardiovascular risk factors^40^. In this study, we observed a tendency of interaction between sex and birth weight for the association with s′, with a positive association observed only in females. As the support for an interaction is weak and multiple outcomes were tested for interactions, this may well be a chance finding. In fact, previous studies have reported no difference in association between FGR and lower s′ according to sex^10^, ^11^.

When compared with previous studies, the novelty and methodological strengths of this study are the combination of well‐defined maternal pregnancy data that allow for better control of confounding, the prospective longitudinal cohort study approach, the duration of follow‐up until adolescence and the use of multiple imputation to address missing data. However, the study also has some relevant limitations. Only a subset of the original birth cohort was examined with echocardiography at a typical age of 17, though a pseudorandom sample was chosen among adolescents attending follow‐up at the time and the birth‐weight *Z*‐score, SBP and BMI were similar. In addition, there were no data on placental dysfunction; hence, our ability to distinguish reduced fetal growth from non‐pathological low birth weight was limited. Furthermore, it should be noted that there are inherent limitations with utilizing estimates of cardiac volumes from two‐dimensional imaging data. For example, it has been reported previously that FGR is associated with left ventricular shape^10^, ^11^. This might affect the geometrical assumptions made when estimating LVMI^10^. Still, our observations for LVMI appeared to be robust across different strategies of accounting for adolescent body size.

In conclusion, higher birth‐weight *Z*‐score across its entire range was associated with minor alterations in diastolic function in adolescence, as measured by echocardiographic indicators. In contrast, the positive association between birth‐weight *Z*‐score and LVMI was driven by those born SGA. All observed effect estimates were small but should be considered in the perspective of life‐course trajectories. Whether and, if so, the extent to which these associations are explained by causal effects mediated through birth weight, pleiotropic genes or suboptimal maternal cardiovascular adaptation to pregnancy^41^ remains to be elucidated.

## Supporting information


**Appendix S1** Methods for data collection and echocardiography examinations in ALSPAC and statistical analyses in current study

**Appendix S2** Results of model‐fit statistics and analyses performed including only participants with complete data and when accounting for height
**Tables S1** and **S2** Association between birth weight and cardiac structure and function in participants without missing birth‐weight or covariable data (Table S1) or with normal birth weight without missing covariable data (Table S2)
**Table S3** Influence of height on association between birth weight and left ventricular mass or left atrial diameter in adolescence
