# Supplementary material for: Birth weight and cardiac function assessed by echocardiography in adolescence: Avon Longitudinal Study of Parents and Children
Source: Ultrasound Obstet Gynecol. 2019 Aug 5;54(2):225–31. doi: 10.1002/uog.20128 (PMC6771817; doi:10.1002/uog.20128)
Supplement: Supplementary file 1 — Appendix S1 Methods for data collection and echocardiography examinations in ALSPAC and statistical analyses in current study [file UOG-54-225-s001.docx]

**Appendix S1** Methods for data collection and echocardiography examinations in ALSPAC and statistical analyses in current study

**SUPPLEMENTAL METHODS**

**Maternal and participant data collection**

We defined preterm birth as <37 weeks gestation. Questionnaires during pregnancy were used to collect data on maternal pre-pregnancy weight, height, maternal level of education (compulsory/vocational, compulsory/higher achievement, secondary/academic preparation, or tertiary/degree), and maternal smoking status in pregnancy (never, stopped prior second semester, smoked during the second semester). Categories of hypertensive disorders of pregnancy (gestational hypertension, preeclampsia/superimposed preeclampsia, and self-reported essential hypertension) were defined using the abstracted data from maternal health records on maternal prenatal blood pressure and dipstick measurements. We defined gestational hypertension as *de novo* hypertension manifesting during pregnancy according to the criteria of the International Society for the Study of Hypertension in Pregnancy^1^ (systolic blood pressure (SBP) ≥ 140 mmHg or diastolic blood pressure (DBP) ≥ 90 mmHg on at least two occasions after the 20th gestational week). Pre-eclampsia was defined as gestational hypertension with concomitant proteinuria based on urine dipstick measurements or proteinuria superimposed on essential hypertension. Pre-existing maternal diabetes mellitus, gestational diabetes mellitus, and glycosuria during pregnancy (according to urine dipstick) were collapsed into one variable.

At the age 17 years clinical assessment, brachial blood pressure and heart rate were measured in a sitting position using an Omron 705-IT machine (typically from the right arm). The mean of two readings was used. Weight was measured in light clothing to the nearest 0.1 kg on Tanita scales (Tanita Europe BV, Amsterdam, Netherlands). Height was measured without shoes to the nearest 0.01 m using a Harpenden stadiometer (Holtain Ltd, Crymych, UK). BMI was calculated as (weight in kg)/(height in m^2^).

**Echocardiography examinations**

Left atrial diameter was measured using M-mode. Tissue Doppler echocardiography was performed in the four chamber view on the lateral left ventricular wall to obtain myocardial wall velocities. RWT was calculated using posterior wall thickness in diastole and left ventricular internal diameter in diastole. Ejection fraction was calculated as the percent of the end diastolic volume that was ejected during each cardiac cycle. s’ was calculated as the average from s’ measurements of the lateral and septal left ventricular wall, each measured in triplicate. The velocity profile for trans-mitral flow was recorded with the patient in passive end expiration. The peak flow velocity of the early (E) and atrial (A) waves in diastole were measured from the three consecutive cardiac cycles displaying the highest measurable velocity profiles. Peak early diastolic (e’) lateral tissue velocity was recorded with a 5 mm sample volume over the mitral valve annulus for 8-10 cycles. The reproducibility of these examinations was assessed by recalling 30 participants and repeating their echocardiographic measurements. The intra-class correlation of repeated measurements ranged from 0.75 to 0.93 (intraobserver) and 0.78 to 0.93 (interobserver).

**Investigating the appropriateness of birth weight as a continuous variable**

We first eyeballed plots of birth weight for gestational age z-scores and each echocardiography outcome. We then investigated the extent to which a dichotomous definition of low birth weight for gestational age (<10^th^ percentile or not) provided a better model fit compared to models with birth weight for gestational age as a continuous variable together (all with adjustment for age and sex). We regarded a difference in Bayes information criterion (BIC) < 6 as evidence in support of comparable model fit.^2^ There was no evidence that the dichotomous categorization was superior. We also examined models in which birth weight for gestational age was included as six categories (<10^th^ percentile, 10-30^th^ percentile, 30-50^th^ percentile, 50-70^th^ percentile, 70-90^th^ percentile, and >90^th^ percentile). Again, there was no evidence that this categorization of birth weight provided better model fit for any of the outcomes compared to models in which birth weight for gestational age was included as a continuum.

**Multiple imputation analysis**

We performed multiple imputation utilizing the mi impute command in STATA. Missing data for the birth weight exposure and all co-variables were imputed using chained equations utilizing all analytical variables. We did not impute data for the echocardiography outcomes, as missing data for these outcomes were typically attributable to failure to estimate the underlying measurements during echocardiography. All imputed variables had <10 % missing data (Table 1) and 20 iterations were used for all imputations. To allow testing for interaction by sex in the main analysis, the imputation datasets were created stratified by sex. All continuous variables were imputed with linear regression. Categorical variables were imputed utilizing the logistic regression method appropriate for each variable depending on number of categories and whether these categories were ordinal. The augmentation option was used to avoid perfect prediction as appropriate. We then analyzed the imputed datasets with the ‘mi estimate’ command utilizing Rubin’s rule, having checked each imputed dataset for large deviations of imputed variables. Evidence of interaction by sex was investigated in the main linear analyses (Model II) for all outcomes.

**Linear spline analyses**

As the association between birth weight for gestational age and LVMI was abrogated when we restricted the birth weight to the 10^th^ to 90^th^ percentiles, we exploratory utilized linear splines to further investigate these results. For these spline analyses we utilized the mkspline command with the marginal option in STATA to test differences in slope of adjacent splines. To focus on the extremes of the birth weight distribution – i.e. to capture fetal growth restriction and those with supra normal birth weight for gestational age – we set spline knots to the 10^th^ and 90^th^ percentile of birth weight. We then tested the change in slope between knots with focus on the extremes of the distribution, adjusting for variables included in Model II.

**SUPPLEMENTAL REFERENCES**

1. Brown MA, Lindheimer MD, de Swiet M, Van Assche A, Moutquin JM. The classification and diagnosis of the hypertensive disorders of pregnancy: statement from the International Society for the Study of Hypertension in Pregnancy (ISSHP). Hypertens Pregnancy. 2001;20(1):IX–XIV.

2. Kass RE, Raftery AE. Bayes Factors. J Am Stat Assoc. 1995;90(430):773–795.
