# Supplementary material for: Birth weight and cardiac function assessed by echocardiography in adolescence: Avon Longitudinal Study of Parents and Children
Source: Ultrasound Obstet Gynecol. 2019 Aug 5;54(2):225–31. doi: 10.1002/uog.20128 (PMC6771817; doi:10.1002/uog.20128)
Supplement: Supplementary file 2 — Appendix S2 Results of model‐fit statistics and analyses performed including only participants with complete data and when accounting for height Tables S1 and S2 Association between birth weight and cardiac structure and function in participants without missing birth‐weight or covariable data (Table S1) or with normal birth weight without missing covariable data (Table S2) Table S3 Influence of height on association between birth weight and left ventricular mass or left atrial diameter in adolescence [file UOG-54-225-s002.docx]

**Appendix S2** Results of model-fit statistics and analyses performed including only participants with complete data and when accounting for height

**SUPPLEMENTAL RESULTS**

There was formal statistical evidence that the null association observed for LVMI in participants with birth weight between the 10^th^ to 90^th^ percentiles was smaller (-2.40 g/m^2.7^ per one z-score unit increase, 95 % CI -4.12; -0.67, p=0.007 for change in slope) than the association observed in those with birth weight <10^th^ percentile (2.44, 95% CI 0.89; 4.00). The association among participants with birth weight >90^th^ percentile, i.e. participants born large for gestational age, was not different from that for participants between 10-90^th^ percentile (1.02, 95 % CI -0.51; 2.55, p=0.19 for difference in slope). No differences by birth percentile were found for any other echocardiography outcome (results not shown). The results of the main analyses did not materially change when we only included participants with complete data (Tables S1 and S2).

The positive association of birth weight with left ventricular mass was robust across different strategies to account for height in the analysis, including adjusting for adolescent height in the regression model as well as indexing by height in m^1.6^ or indexing by body surface area (Table S3). The association with left atrial diameter appeared more sensitive to the handling of height in the analysis. In some contrast to our main analysis, there was an inverse association between birth weight and indexed left atrial diameter to height^1.6^, height^2.7^, or body surface area also in model II (Table S3).

| **Table S1** Association between birth weight and cardiac structure and function in participants without missing birth-weight or covariable data | | |
| --- | --- | --- |
|  | **Model** | ***β*-coefficient (95% CI)** |
| **Cardiac structure** |  |  |
| Left ventricular | I | 0.42 (0.12, 0.72) |
| mass index, g/m^2.7^ | II | 0.38 (0.07, 0.70) |
| (N=1,591) | III | 0.04 (-0.23, 0.31) |
|  |  |  |
| Left atrial diameter | I | -0.003 (-0.010, 0.015) |
| index, cm/m | II | -0.002 (-0.015, 0.011) |
| (N=1,426) | III | -0.014 (-0.026, -0.003) |
|  |  |  |
| Relative wall thickness | I | 0.0005 (-0.0022, 0.0033) |
| (N=1,591) | II | 0.0009 (-0.0020, 0.0038) |
|  | III | 0.0006 (-0.0023, 0.0035) |
| **Systolic function** |  |  |
| Ejection fraction, % | I | -0.26 (-0.56, 0.05) |
| (N=1,592) | II | -0.29 (-0.62, 0.04) |
|  | III | -0.25 (-0.58, 0.08) |
|  |  |  |
| s’ | I | 0.05 (-0.02, 0.13) |
| (N=1,522) | II | 0.04 (-0.04, 0.12) |
|  | III | 0.04 (-0.04, 0.12) |
| **Diastolic function** |  |  |
| E/A | I | -0.023 (-0.042, -0.003) |
| (N=1,533) | II | -0.021 (-0.042, -0.0003) |
|  | III | -0.022 (-0.043, -0.001) |
|  |  |  |
| E/e’ | I | -0.07 (-0.12, -0.02) |
| (N=1,520) | II | -0.06 (-0.11, -0.003) |
|  | III | -0.04 (-0.09, 0.01) |
| CI: Confidence Interval  Birth weight is modelled as standardized birth weight per gestational week and sex.  Model I: adjusted for age at examination and sex (male or female)  Model II: additionally adjusted for: maternal height, maternal hypertensive disorders of pregnancy (preeclampsia, gestational hypertension, essential hypertension, or none), maternal age at pregnancy, maternal BMI, maternal parity (parous or nulliparous), maternal smoking during pregnancy (never, stopped prior second semester, smoked during the second semester), maternal education (compulsory/vocational, compulsory/higher achievement, secondary/academic preparation, or tertiary/degree), maternal diabetes mellitus during pregnancy (yes or no), and preterm birth (<37 weeks, yes or no)  Model III: additionally adjusted for factors in adolescence: body mass index, systolic blood pressure, and heart rate | | |

| **Table S2** Association between birth weight and cardiac structure and function in participants with normal birth weight without missing covariable data | |
| --- | --- |
|  | ***β*-coefficient (95% CI)** |
| **Cardiac structure** |  |
| Left ventricular mass index, g/m^2.7^ (N=1,280) | 0.03 (-0.48, 0.54) |
| Left atrial size index, cm/m (N=1,159) | -0.003 (-0.024, 0.018) |
| Relative wall thickness (N=1,280) | 0.0008 (-0.006, 0.004) |
| **Systolic function** |  |
| Ejection fraction, % (N=1,281) | -0.39 (-0.92, 0.14) |
| s’ (N=1,227) | 0.10 (-0.03, 0.22) |
| **Diastolic function** |  |
| E/A (N=1,234) | -0.03 (-0.06, 0.005) |
| E/e’ (N=1,226) | -0.08 (-0.17, 0.01) |
| Birth weight is modelled as standardized birth weight per gestational week and sex. Normal birth weight is defined as birth weight z-score corresponding to the 10th to 90th percentile.  Models (II) adjusted for age at examination, sex (male or female), maternal height, maternal hypertensive disorders of pregnancy (preeclampsia, gestational hypertension, essential hypertension, or none), maternal age at pregnancy, maternal BMI, maternal parity (parous or nulliparous), maternal smoking during pregnancy (never, stopped prior second semester, smoked during the second semester), maternal education (compulsory/vocational, compulsory/higher achievement, secondary/academic preparation, or tertiary/degree), maternal diabetes mellitus/glycosuria during pregnancy (yes or no), and preterm birth (<37 weeks, yes or no) | |

| **Table S3** Influence of current height on association between birth weight and left ventricular mass or left atrial diameter in adolescence | | | | |
| --- | --- | --- | --- | --- |
|  | **Birth weight by GA** | | | |
|  | **Unrestricted** | | **10^th^ to 90^th^ percentile** | |
|  | ***β*-coefficient (95% CI)** | **P-value** | ***β*-coefficient (95% CI)** | **P-value** |
| **Left ventricular mass, g** |  |  |  |  |
| Non-indexed | 4.88 (3.61, 6.15) | <0.001 | 3.35 (1.31, 5.38) | 0.001 |
| Non-indexed, adjusted for height | 3.33 (2.04, 4.63) | <0.001 | 1.87 (-0.17, 3.92) | 0.07 |
| Indexed to m^1.6^ | 1.24 (0.73, 1.76) | <0.001 | 0.59 (-0.24, 1.42) | 0.17 |
| Indexed to BSA | 1.17 (0.57, 1.76) | <0.001 | 0.27 (-0.69, 1.24) | 0.58 |
|  |  |  |  |  |
| **Left atrial diameter, cm** |  |  |  |  |
| Non-indexed | 0.020 (-0.0021, 0.039) | 0.05 | 0.022 (-0.010, 0.053) | 0.18 |
| Non-indexed, adjusted for height | 0.016 (-0.005, 0.036) | 0.14 | 0.018 (-0.014, 0.051) | 0.26 |
| Indexed to m^1.6^ | -0.013 (-0.022, -0.004) | 0.006 | -0.014 (-0.028, 0.0003) | 0.06 |
| Indexed to m^2.7^ | -0.015 (-0.021, -0.009) | <0.001 | -0.017 (-0.026, -0.008) | <0.001 |
| Indexed to BSA | -0.028 (-0.039, -0.017) | <0.001 | -0.031 (-0.049, -0.014) | <0.001 |
| CI: Confidence Interval; GA: Gestational age  Birth weight is modelled as standardized birth weight per gestational week and sex.  All models includes age at examination and sex (male or female), maternal height, maternal hypertensive disorders of pregnancy (preeclampsia, gestational hypertension, essential hypertension, or none), maternal age at pregnancy, maternal BMI, maternal parity (parous or nulliparous), maternal smoking during pregnancy (never, stopped prior second semester, smoked during the second semester), maternal education (compulsory/vocational, compulsory/higher achievement, secondary/academic preparation, or tertiary/degree), maternal diabetes mellitus during pregnancy (yes or no), and preterm birth (<37 weeks, yes or no) | | | | |
